# The Institutional Library

**Published:** 1919-06-07

**Authors:** 


### THE INSTITUTIONAL LIBRARY.

**The Doctor in War.** By WOODS HUTCHINSON, M.A., M.D. (Cassell and Co., Ltd., London, New York, Toronto and Melbourne. Pp. 299. 1917. Price 7s. 6d.)

THE author of this attractive volume spent the year 1917 in visiting the medical war establishments of Britain, America, France and Italy, from the bases to the firing line, and has succeeded in presenting to the public a vivid word-picture of one of the most marvellous achievements in the history of the world—the triumph of the doctor. In proportion to the numbers engaged, the Greatest of Wars has been much the least dangerous of which we have any record. The average death-rate of the first three great wars of the past century—the Napoleonic, the Mexican and the Crimean—was 12 per cent. to 5 per cent. a year; that of the last three wars—the Spanish-American, the Boer and the Russo-Japanese—was 4 per cent. to 8 per cent., and that of the Great War from 2 per cent. to 3 per cent.

The most influential factor in this improvement is convincingly ascribed to the advance in medical science. The fatality from wounds has been tremendously reduced. There were as many wounded in proportion to killed as before, only instead of 20 per cent. to 50 per cent. dying from their wounds as in the old days there were only 5 per cent. to 10 per cent., 90 per cent. of the wounded were also saved, and of these 80 per cent. returned to the firing-line within forty days. Disease as a factor in the army death-rate has been almost wiped out, completely so in the sense that the amount of sickness and the deaths from disease at the front have been barely half what they were in barracks in times of peace. The main causes of this transformation are dealt with in separate chapters. The perils of war, medical and surgical, are described in a popular and interesting style, also the means of prevention, including general hygiene and personal prophylaxis. The triumph over "typhoid," with its associated paratyphoids, the scourges of previous war, by a triple vaccine, aided by the search for, and isolation of "carriers," a pure water supply, and the campaign against flies is described in detail. The new diseases, trench fever, trench foot, and trench nephritis, are given a separate chapter, which is full of interest, and the victory over dysentery, scurvy and cholera, the slayers of thousands in previous wars, is recorded. The application of science to the prevention of these and other diseases involved the co-operation of every department of the army on a stupendous scale; not the least important being the supply and transport of food and water. The soldier was generously fed, the ration including white bread, beef, fat, sugar and vegetables, and tea instead of alcohol. The water supply and its purification and distribution was even more remarkable than that of food. During the long period of trench warfare the troops were served on a communal standard, pipe lines, many miles in length, were laid from pure sources up to tanks placed at intervals in the trenches, each provided with a long row of taps. Wells, however, had to be used frequently, and in Northern France even the best of them were contaminated by either privy content or the intensely manured soil, a danger which is met by a meticulous system of chlorination. During an advance the British employed a motor water convoy, one of the marvels of the war. Its objective is rapid clarification and chlorination; the unit consists of a group of tanks with an engine and purifying system mounted on a large motor truck; the progress of filthy pea-soupy water through the various processes of filtration, precipitation and chlorination to clear sparkling fluid, at the rate of 1,000 gallons an hour, reads like a fairy tale. The failure of pre-war aseptic surgery to meet the requirements of wounds contaminated by the intensely manured soil of Northern France, teeming with the germs of tetanus, gas gangrene and suppuration, gradually led to the development and building up of a more appropriate system; dead and damaged tissues were excised, and the exposed cavities and pockets treated by the Carrel system of fractional irrigation or intermittent flushing until bacteriological examination indicated sterilisation and the time for secondary suture, a method which was responsible for the saving of many lives and limbs.

The book is a record of the personal experiences of an enthusiast which covered a very wide field of inquiry; every chapter is equally interesting, the chapters on "war gas," shell shock, the troubles of aviators, the fashioning and building of new faces and jaws, and the casualty clearing-station particularly so. We should have appreciated reference to the glorious work of the British Red Cross Society, to the elaborate arrangements for the prevention of surgical shock, and to the great advance in anæsthetics, each of which has played such an important part in the "triumph of the doctor"; we cannot, however, expect a complete treatise in a volume of 300 pages. The book is illustrated by excellent photographs, and it should appeal to a large public, and equally to doctors and nurses.

**Fevers in the Tropics.** By Sir LEONARD ROGERS, C.I.E., M.D., etc., Lieut.-Colonel, I.M.S. Third Edition. (Henry Frowde and Hodder and Stoughton. 1919. Price 30s. net. Pp. 404.)

The second edition of this valuable book followed so hard on the heels of the first that but little opportunity for expansion or revision could be accepted. It is, however, now ten years since the original issue; and Sir Leonard Rogers has so extensively incorporated the many and profound advances in the knowledge of tropical fevers which have been made during recent years that this edition almost amounts to a new text-book. Beginning with kala-azar, a plague which Sir Leonard has long made peculiarly his own especial study, the reader is conducted through the trypanosomiases, the enterics, and other prolonged fevers, to the malarias, dengue, plague, and yellow fever, and then to heatstroke, and so to a few closing remarks on the exanthemata as seen in the tropics.

A book full of such profound clinical wisdom, combined with so varied a scientific knowledge of all the marvellous advances of recent years in tropical diseases—advances many of which have been made or led by the author himself—has perhaps never before appeared. Only a generation ago a book of this scope would have been held to render all rivals unnecessary for many years; but as matters stand it will doubtless not be long before research once more outstrips the text-book. In the whole volume there is but one section which seems a trifle "scrappy"—only by comparison with the rest of the book *bien entenu*—and that is the account of the paratyphoid fevers. But of the book as a whole it is impossible to do other than express the most whole-hearted admiration and contentment. The publishers, too, deserve a word of commendation for the beautiful format of the volume, which reflects the highest credit on them as well as upon the distinguished author.

**Institutional Library**—(*continued*).

**Massage as a Career for Women.** By Beatrice M. Goodall-Copestake, Examiner to the I.S.T.M.'s Teacher of Massage and Remedial Exercises to the Nursing Staff of the London Hospital. (London: George Newnes, Ltd. Price 9d.)

This modest brochure well deserves its description as "an up-to-date and invaluable booklet," though it might well have been praised in a less illiterate manner. It testifies to complete mastery of the subject, and embraces all that is needed to place the masseuse in close relation with the chosen profession. It gives a clear account of the different training centres, the cost and duration of the various courses open to the learner, and the openings and probable income awaiting the trained woman. The chapter on Massage in Military and Naval Hospitals and in Private Practice is particularly interesting.

**The Cost of Food:** A Study of Dietaries. By Ellen H. Richards, late Instructor in Sanitary Chemistry, Massachusetts Institute of Technology. Third Edition. Revised under the direction of John T. Norton, Ph.D. (New York and London: Chapman and Hall. Price 5s.)

Mrs. Richards' very sensible manual on foodstuffs, their cost and comparative value, was issued in 1908 and has achieved well-deserved popularity. It has been revised only where present-day ideas are radically different from those which prevailed at that date, but already, as regards prices, it is far behind, the figures selected being those of the years 1915-16. The valuable part of the book is its recognition of the varying needs of individuals in respect of food. There are admirable chapters on school dietaries, food for the professional person, food for hospitals, food for old age. The remarks on "Malnutrition in Middle Life" are the best in the book.

**Manual of Bacteriology.** By Robert Muir, M.A., M.D., Sc.D., F.R.S., and James Ritchie, M.A., M.D., F.R.C.P.(Ed.). Seventh Edition. (London: Henry Frowde, Oxford University Press, and Hodder and Stoughton, Warwick Square, E.C. 16s. net.)

In this new edition particular note has been made of many results of that impetus given to bacteriology by the urgent requirements of practical medicine and surgery during war. Considerable enlargement and addition has been made to the chapters dealing with cerebrospinal fever, intestinal infection, both bacterial and protozoal in nature, tetanus and the variety of conditions arising in septic wound complications. New sections appear concerning varieties of infective jaundice and trench fever. Also in the general revision which has been made, a number of new technical descriptions are introduced and points of future development indicated. On the whole one may say that, in spite of the tremendousness of the task in keeping such a manual abreast of rapidly advancing research, those responsible have brought it to a stage of such perfection that it maintains its position as one of the most valuable of books to both the practical bacteriologist and the physician.

**Pye's Surgical Handicraft.** Edited and largely re-written by W. H. Clayton-Greene, M.B., B.C., F.R.C.S.

This well-known volume has been brought further up to date as a result of the frequent and necessary surgical advances during the war period. Many chapters have been rewritten, new illustrations added, and a considerable amount of new matter appears. The treatment of orthopœdics is followed on the classic lines of Sir Robert Jones' teaching, and makes a most valuable résumé of this work.

**Bodily Deformities.** By the late E. J. Chance, F.R.C.S. Edited by John Poland, F.R.C.S. Vol. II. On the Treatment of Deformities. (Published by John Murray, London, W. 18s. net.)

This volume comprises a further series of lectures upon a subject which will appeal to an increasing circle of surgical readers. The editor has provided useful and copious notes, with an excellent series of illustrative plates from cases in his own practice. The whole makes a highly interesting survey of the subject from both a practical and historical point of view.

**Handbook of Physiology.** By W. D. Halliburton, M.D., F.R.S. Fourteenth edition. (London: John Murray. Price 16s. net.)

So short a time has elapsed since the publication of the previous edition of this book that any extensive alteration has been found unnecessary. Attention may be drawn to a highly interesting chapter on War Diet, which appears as an appendix, and which may well interest all who have taken or may in future take part in service operations, or find themselves in war-stricken countries. One notes that in accordance with the recommendation of the Anatomical Society of Great Britain and Ireland the old nomenclature has been resumed, and, we think, advisedly. Of the volume itself one need say no more than that the present edition maintains the excellent standard of its predecessors.

**Elements of Surgical Diagnosis.** By Sir Alfred Pearce Gould. Fifth edition, revised. (Published by Cassel and Co. Price 12s. 6d. net.)

This small volume remains to a great extent in the form which has long been known. It has, however, been brought up to date in the practical as well as the diagnostic sense. Much new matter has been introduced, such as the details of gas gangrene infections, etc., and omission of several older methods of diagnosis, rendered obsolete by the extended use of X-rays and laboratory processes, has tended greatly to clear the view of the surgical art with which the student is provided. The revision has resulted in need for slightly less pages than before, in spite of the increased information contained; and, as the authors state in their preface, this is a not unwelcome, if unusual, feature of a new edition of a book intended primarily for students.

**Kala-Azar: its Diagnosis and Treatment.** By Ernest Muir, M.D. With a Foreword by Sir Leonard Rogers, C.I.E., I.M.S. (Calcutta: Butterworth and Co., Ltd. 1918. Pp. 37 + v. Price R. 2 net.)

The story of the investigation of tropical diseases is one of the triumphs of modern medicine, and it emphasises, what is taught also from other sources, that the basis of successful treatment and efficient prevention is the discovery of causes. In this unpretending manual the author confines himself to the strictly practical aim of describing the diagnosis and treatment of one of these diseases, namely, Kala-Azar. The death-rate in this disease is about 80 per cent., and the sufferers in certain parts of India are numerous. Provided the condition is recognised sufficiently early, it can almost certainly be cured by the intravenous injection of soluble antimony salts. Clearly this knowledge ought to be in the hands of all practitioners in the districts concerned, and to these, as well as to the sufferers, Dr. Muir's exact and detailed description is a valuable and helpful contribution. It is animated by a steady purpose and is well adapted to achieve the end which it proposes. The illustrations increase the efficiency of what is in all respects a really useful handbook.

